# Proteins and Metabolites as Indicators of Flours Quality and Nutritional Properties of Two Durum Wheat Varieties Grown in Different Italian Locations

**DOI:** 10.3390/foods9030315

**Published:** 2020-03-09

**Authors:** Sara Graziano, Nelson Marmiroli, Giovanna Visioli, Mariolina Gullì

**Affiliations:** 1 Interdepartmental Center SITEIA.PARMA, University of Parma, Parco Area delle Scienze, 43124 Parma, Italy; sara.graziano@unipr.it (S.G.); nelson.marmiroli@unipr.it (N.M.); 2 Department of Chemistry, Life Sciences and Environmental Sustainability, University of Parma, Parco Area delle Scienze 11/a, 43124 Parma, Italy; giovanna.visioli@unipr.it; 3 National Interuniversity Consortium for Environmental Sciences (CINSA), Parco Area delle Scienze 17, 43124 Parma, Italy

**Keywords:** gliadins, glutenins, starch, polyphenols, antioxidant capacity

## Abstract

Durum wheat is an important food source in Mediterranean countries, and Italy is the major producer of durum wheat in Europe. The quality of durum wheat flours depends on the type and amount of gluten proteins and starch while flour nutritional value rests on metabolite contents such as polyphenols. In this work, two Italian cultivars, Iride and Svevo, were analyzed for two years (2016–2017) in four Italian regions to explore how the environment affects: (i) reserve proteome; (ii) starch content and composition; and (iii) free, conjugated, bound phenolics and antioxidant capacity. The impact of environmental and meteorological conditions was significant for many traits. Regardless of the cultivation site, in 2017, a year with less rainfall and a higher temperature during grain filling, there was an increase in low molecular weight glutenins, in the glutenin/gliadin ratio and in the A-type starch granules size, all parameters of higher technological quality. In the same year, the cultivars showed higher amounts of polyphenols and antioxidant capacity. In conclusion, the two wheat cultivars, selected for their medium to high yield and their good quality, had higher performances in 2017 regardless of their sowing locations.

## 1. Introduction

Durum wheat (*Triticum turgidum* L. subsp. durum (Desf.) Huns) is an important food source with a relevant role in the human diet, providing carbohydrates, proteins and energy to the human diet [1,2]. Italy was the first producer of durum wheat within Europe, and Italians have the highest *pro capite* consumption rates of pasta in the world. Therefore, great effort has been utilized over the years to select durum wheat genotypes with the best agronomic and technological performances [3]. Seed protein content and composition are important factors influencing flour quality [4]. Wheat kernel contains different types of proteins, classified as albumins/globulins and gluten proteins. Albumins/globulins are non-storage proteins involved in germination metabolic processes which represent about 20% of seed proteins, while gluten proteins, which represent 80%, determine the elasticity and extensibility of dough, which are important technological properties [5]. Gluten proteins are divided into two classes: (i) gliadins, alcohol-soluble proteins represented by α/β, γ, and ω-fractions, in relation to their electrophoretic mobility; and (ii) glutenins, low molecular weight (LMW-GS) and high molecular weight (HMW-GS) subunits, organized in complex heteropolymers, which are stabilized by both intermolecular and intramolecular disulfide bonds [6]. In addition to gluten proteins, wheat seeds contain a variable amount of starch, from two-thirds to three-quarters of the dry weight of the seed, depending on the cultivar. [7,8]. Starch characteristics may influence some technological parameters, such as pasting properties, gelatinization, and retrogradation, which are important in food making and in industrial applications [9]. Starch is organized into two different types of granules: A-type (large, lenticular, diameter of 10–35 µm); and B-type (small, spherical, diameter of 1–10 µm) with different physical, chemical, and functional properties [10].

A-type and B-type granules contribute >70% and <30% of the total starch weight, respectively, and account for about 10% and >90% of the total granule number, respectively [7]. The B-granules vary based on the genotype, environment, and method of analysis [11]; moreover, an increase in the B-type granules content improves pasta quality [9].

Environmental stress affects the quantity and quality of the reserve proteins and starch accumulation in wheat kernels [12,13], as well as the genotype [14]. In Mediterranean environments, water scarcity, which is often associated with high temperatures during flowering and grain filling, becomes a main factor influencing the durum wheat yield as well as the protein quality and quantity [15]. It is widely accepted that the genotype, growing environment, and their interaction affect both the quality traits and the nutrient composition of wheat [14,16]. Heat stress and water deficit during growth also cause a significant reduction in starch content, which may reflect the kernel weight and diameter [17], increasing the pasting temperature and causing higher viscosity of the dough [18,19].

Wholegrains are also a source of important antioxidants, such as polyphenols and vitamins, and full characterization of nutraceutical properties in wholegrains is important in the branding and marketing of wheat [20]. It has been demonstrated that a diet based on wholegrains and their products may reduce the incidence of chronic diseases, and these benefits may depend on the phytochemicals contained in wheat grain. In recent years, interest in the study of polyphenols has increased, as they exert antioxidant activity, and a correlation between the amount of polyphenols and the antioxidant activity has been shown [21]. Several studies have shown that the composition of phenolic acids varies extensively in cereals [2]. Polyphenols are synthetized in response to external factors; therefore, their amount is strongly influenced by environmental constraints [22]. Biosynthesis and accumulation of phenolic compounds during kernel development are highly dependent on the wheat variety and environmental conditions [3,23]. 

This work reports a comparison of the flour quality of two *T. durum* cultivars (cvs): Iride and Svevo, grown in four Italian regions with different pedoclimatic conditions, in two growing years. In particular, the analysis involved: (i) the content and composition of storage gluten proteins; (ii) the amount of starch granules, both A and B-types; (iii) the amount of phenolic acids and of their antioxidant properties. The differences observed are discussed in relation to both genetic and environmental factors. 

## 2. Materials and Methods

### 2.1. Plant Material and Field Trials 

Two cvs of durum wheat (*Triticum durum* Desf.) were utilized in this study: Iride and Svevo both of which were released in 1996 by PSB s.p.a. (Italy). Iride is one of the most cultivated durum wheat cv in Italy, in particular, for the exceptional fertility of the ear and the excellent environmental adaptability, whereas Svevo has an extraordinary aptitude for industrial transformation. The main characteristics of the two cvs are shown in more detail in Table 1. 

Plants were grown in the Italian durum wheat variety trials “Rete nazionale duro” in two consecutive growing seasons (2015–2016 and 2016–2017, indicated from now on as 2016 and 2017, respectively) in four locations: Argelato (BO) (44°39’ N, 11°20’ E, 25 m a.s.l.), Tolentino (MC) (43°14’ N, 13°24’ E, 184 m a.s.l.), Foggia (FG) (41°27 ’ N, 15°30’ E, 76 m a.s.l.), and S. Stefano Quisquina (AG) (37°32’ N, 13°31’ E, 180 m a.s.l.). The agronomic management followed a standard protocol for the Italian durum wheat variety trials. In each environment, rainfall distribution and temperatures were recorded during the 2016 and 2017 cropping seasons and are available within the national trial dataset. 

All grain samples were collected after harvesting and kindly provided by the Council for Agricultural Research and Economics, Research Centre for Cereal and Industrial Crops (CREA-CI) of Foggia (Italy).

### 2.2. Seed Storage Protein Extraction and Quantification

Gluten proteins (gliadins, HMW-GS and LMW-GS), were extracted from wholemeal wheat semolina (30 mg) using a previously described sequential extraction procedure [1]. The amounts of extracted gliadins and glutenins fractions were determined with the Bradford assay (Bio-Rad, Hercules, CA). The amount of each fraction was calculated in relation to the total amount of extracted storage proteins. The grain protein content (GPC%) was measured as previously reported [14,24]. 

### 2.3. Protein Characterization by Sodium Dodecyl Sulfate Polyacrylamide Gel Electrophoresis (Sds-Page) and Densitometric Analysis of Gluten Fractions 

SDS-PAGE was performed in a Mini-PROTEAN Tetra Cell (Bio-Rad) using 12% for the LMW-GS and gliadins, and 8% for the HMW-GS of acrylamide gels, as previously reported [24]. LMW-GS, HMW-GS, and gliadins (20 μg each) were suspended in 20 μL of loading buffer. The Low Molecular Weight Calibration Kit for SDS Electrophoresis (MW 14,400–97,000 Da) (GE, Healthcare, Chicago, Illinois, USA) was used to detect bands. After electrophoretic separation, the gels were fixed in 70 mL/L acetic acid and 400 mL/L methanol and stained with Coomassie Brilliant Blue R-250 Staining Solution (Bio-Rad). Protein molecular weights (MWs) and the relative quantification of the LMW-GS, HMW-GS, and gliadin bands were obtained using the Image Lab 4.5.1 software (Bio-Rad). Gliadins were subdivided into two classes (ω- and α-, γ-) on the basis of their molecular weights, as previously reported [25]. In order to compare each fraction composition, the relative amounts of sub-fractions were calculated by considering all bands present in each lane.

### 2.4. Isolation of Starch

Wheat starch was isolated from wholemeal semolina by using the protocol described by Budney and colleagues [26] with some modifications. Briefly, bran fractions with a particle size greater than 500 µm were discarded. Then, 2 g of wholemeal semolina was mixed with 3 mL of water to form a round piece of dough, which was then hydrated in water for 20 min. Starch was isolated using 80 mL of water. The starch slurry was filtered through a 70 µm sieve to obtain a non-purified liquid fraction with prime starch, while non-starch particles with a size greater than 70 µm remained on the sieve.

The liquid fraction was cooled to 3 °C for 24 h and centrifuged at 2300× *g* for 10 min at 4 °C. The pellet was then subjected to a fractionation procedure. From the pellet, the upper, loosely-packed slower sedimenting layer, which is characterized by a pale yellow-brownish color, was carefully removed with a spatula. The white main starch fraction was purified twice with 20 mL of water and centrifuged at 2300× *g* for 10 min at 4 °C. The starch sample was dried at 40 °C for 24 h.

### 2.5. Light Microscopy

Starch granules were visualized using an AXIO Image Z2 (Zeiss, Jena, Germany). Sample powder (10 µg) was sprinkled on a glass slide and covered with a single drop of an aqueous glycerol solution (1:1 water/glycerol) and covered with a coverslip. Micrographs were obtained at 400× magnification. Four slides were prepared for each sample, and three micrographs were selected from a total of ten for each sample, counting a total of 500 granules of starch. The granule area was measured using ImageJ software (version 1.50i, https://imagej.nih.gov/ij/ open source program last accessed December 2019), and the diameter of starch granules was computed using the Excel program. The numbers of A-type and B-type starch granules were determined while assuming that starch granules had spherical shapes as a function of their diameter (<10 μm and >10 μm).

### 2.6. Determination of Phenolic Acid Content

The assay was performed as previously described [14]. In particular, phenolic acids were quantified using a modified Folin–Ciocalteau assay: 10 μL of sample was added to 95 μL of Folin–Ciocalteau reagent (Sigma-Aldrich), previously diluted to 1:10; then, the reaction was neutralized with 20% sodium carbonate. After 5 min of incubation at 22 °C and 30 s of agitation, the absorbance was read at 750 nm using the iMark microplate reader (Bio-Rad). Gallic acid was used as a standard (6.25−100 μg/mL), and the total phenolic content was expressed as μg/g of gallic acid equivalent (GAE). 

### 2.7. Determination of Antioxidant Activity 

The 2,2’-azino-bis-(3-ethylbenzothiazoline-6-sulfonate) radical cation (ABTS^•+^) scavenging capacity was performed according to Re et al. [27] to determine the antioxidant activity. Briefly, ABTS^•+^ was produced by adding 2.45 mM of potassium persulfate to 7 mM of ABTS^•+^ solution and allowing the mixture to react in the dark at room temperature for 16 h. Then, the solution was diluted 1:80 with methanol to obtain an absorbance of 0.70 ± 0.02 OD at 735 nm. Fresh ABTS^•+^ solution was prepared for each assay. One hundred microliters of sample were added to 1 mL of ABTS^•+^ solution (A_735nm_ 0.70 ± 0.02), and the A_735nm_ was read after 5 minutes. Trolox was used as a standard (25–250 µM), and the determination of antioxidant activity was expressed as µM/g of Trolox equivalent (TE).

### 2.8. Statistical Analysis

Statistical analyses were performed using IBM SPSS Statistics for Windows, Version 25.0. (IBM Corp., Armonk, NY, USA). All data presented are the means of three replicates. For the evaluation of the storage protein amounts, densitometric analysis of bands, starch and antioxidant properties, first, we performed an analysis of variance (ANOVA). Then, all parameters were evaluated together by a multivariate analysis (MANOVA). Whenever a statistically significant effect was detected, the post-hoc Dunn’s test was applied to identify the differences among the pairs of samples. 

The amounts of the different gliadins, LMW-GS, and HMW-GS sub-units, which were calculated as reported in Section 2.3, are shown as heat maps, and were obtained with Heatmapper software [28].

## 3. Results and Discussion

### 3.1. Meteorological Conditions in the Two Growing Seasons 

The four growing environments had very different meteorological conditions [14,24]. With the exception of January, more abundant and more evenly distributed rainfall occurred in 2016 as compared with 2017. This trend was more evident during the vegetative period and during grain filling when rainfall from the second ten-day period of April (flowering) to the second ten-day period of June (harvest) was higher in 2016 with respect to 2017 (181 mm vs. 55 mm). In all locations, the Tmax during the grain filling period was higher in 2017 than in 2016, with more days exceeding 30 °C in most of the locations (Table S1). 

It is well known that high temperatures during seed development represent a stressful condition which affects the duration and rate of grain filling [14,29,30]. Indeed, for many wheat varieties, a few days with a maximum temperature over 30 °C can result in lower quality traits such as flours with weaker dough properties [31,32,33].

The amount of rain was also recorded in all locations in the two growing seasons, and some differences were observed between the two years, mainly in the reproductive period (Table S1). Plant development during vegetative growth is positively influenced by the water availability, while water excess or scarcity during grain filling may change the gluten protein quantity [34]. 

### 3.2. Effect of Cultivar, Growing Area, and Cropping Season on Grain Quality and Nutritional Properties

#### 3.2.1. Analysis of Gluten Protein Content 

The gluten protein content (GPC %) was evaluated in both cvs in the two years in all locations. Each cv had comparable GPC values in the four locations (Table 2), with the exception of Quisquina for which the highest GPC values (*p* < 0.05) were observed (>15%). Overall, Svevo had a significantly higher (*p* < 0.05) GPC than Iride (Table 2). It is well documented that protein content is determined by several major genes and quantitative trait loci (QTLs), but it is also known that each cv has different alleles accounting for the amount and quality of gluten proteins [35,36]. 

The accumulation of gluten protein is a complex process undergoing spatial and temporal regulation, which is affected by environmental conditions [6,37]. Synthesis of gluten proteins occurs from about 8 until 35 days after anthesis, with gliadins being accumulated faster than glutenins. Consequently, the glutenin/gliadin (glu/gli) ratio decreases progressively during grain development [25,38].

It was reported that the protein content may vary due to stressful environmental conditions during grain filling, affecting both kernel size and composition [39,40]. 

Therefore, the amount of each gluten protein fraction was compared in Iride and Svevo cultivated in 2016 and 2017 in the four locations. As expected, the gliadins fraction was the most abundant component, followed by the LMW-GS and the less abundant HMW-GS (Table 2). The HMW-GS amount was generally stable over the two years in both cvs with no significant values among location, year, or cv. The variations in the amount of gliadins and LMW-GS in both cvs was significantly influenced by the cropping season (Table 2 and Table 3). In particular, in 2017, gliadins were significantly lower (*p* < 0.05) in the four locations as compared to in 2016 (Table 3); conversely, LMW-GS increased significantly (*p* < 0.05). Due to the differences in these two gluten fractions, the corresponding glu/gli and HMW-GS/LMW-GS ratios, which are important quality parameters [41], varied significantly with both the year and the cultivation area (Table 3). The glu/gli ratio increased in 2017 as compared to 2016, while the HMW-GS/LMW-GS ratio decreased due to the higher amount of LMW-GS. These results are in agreement with the high quality of these two Italian cvs in 2017 as indicated by Italian crop quality reports [42,43]. In addition, several authors have shown how a well-developed gluten network would preferentially involve a higher amount of LMW-GS as compared with HMW-GS in the assembly of the largest gluten complexes, determining a better dough quality [44,45,46]. No differences between the two cvs were observed in the trend of the gluten fractions in the two years (Table 3). 

#### 3.2.2. Quantification of Gluten Protein Sub-Units 

**Figure 1:**
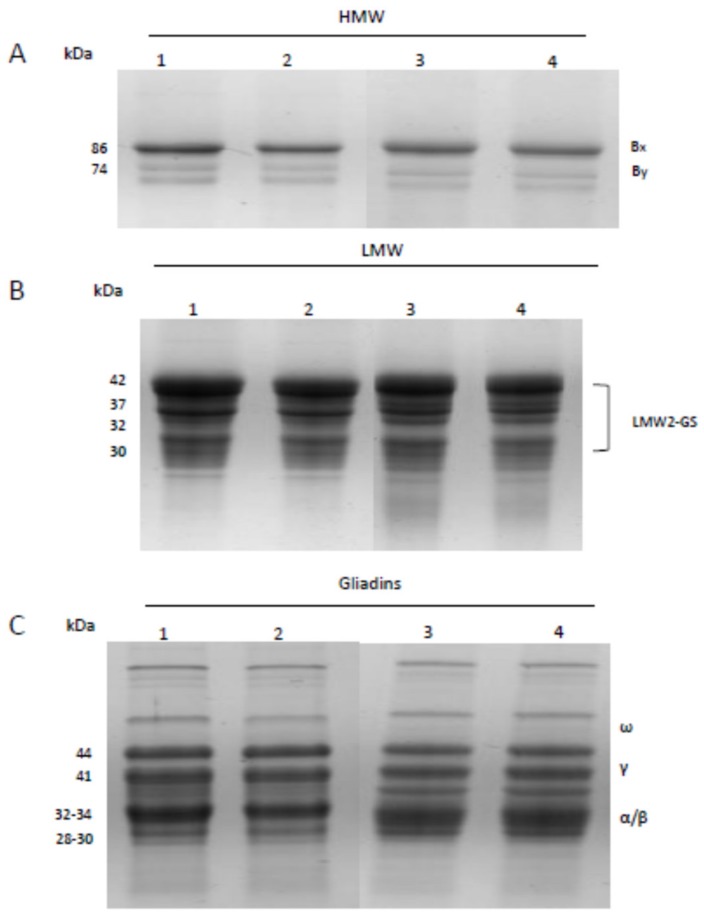
Electrophoretic analysis of gluten proteins by sodium dodecyl sulfate polyacrylamide gel electrophoresis (SDS-PAGE). The pictures show an example of the banding patterns of (**A**) high molecular weight glutenin (HMW-GS), (**B**) low molecular weight (LMW-GS) and (**C**) gliadins purified from Svevo (1–2) and Iride (3–4).

**Figure 2:**
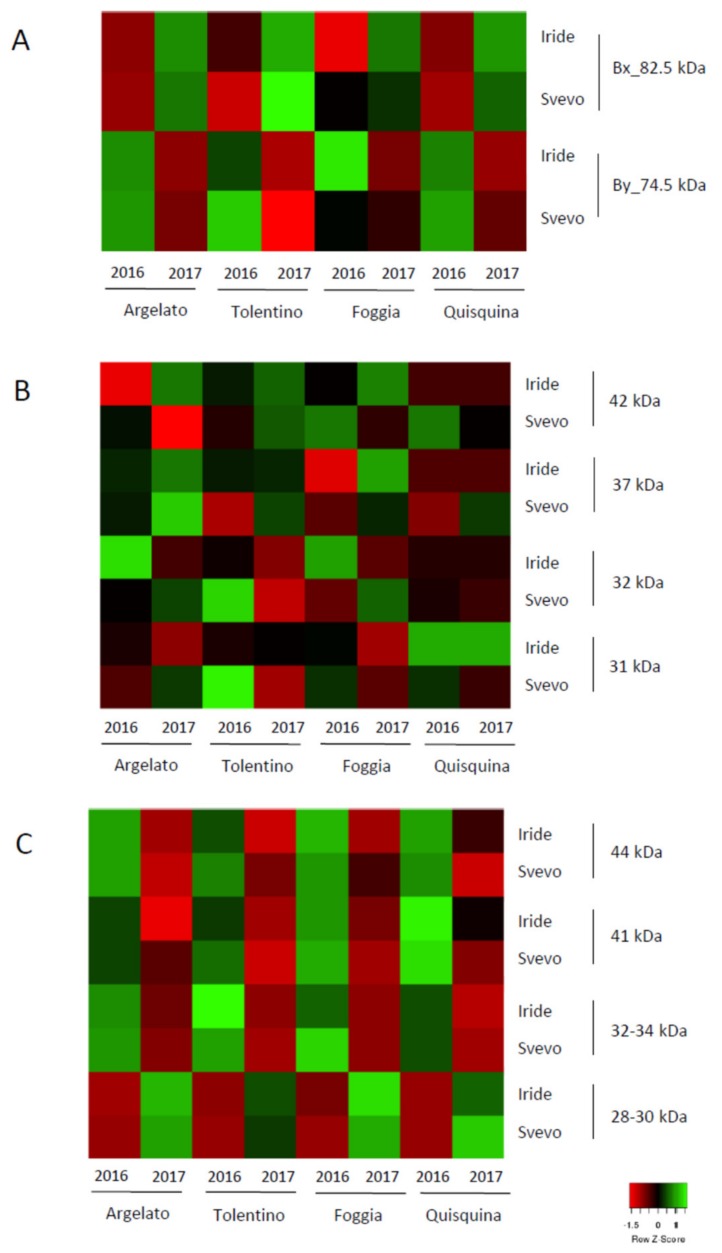
Heat map representing the relative abundance evaluated by densitometric analysis of HMW-GS (**A**), LMW-GS (**B**), and gliadins (**C**) purified from flours of Iride and Svevo cultivated in 2016 and 2017 in four Italian regions.

All gluten fractions were analyzed in both cvs by SDS-PAGE, and Figure 1 shows an example of the profiles of HMW-GS, LMW-GS, and gliadins. As expected, Iride and Svevo have a similar banding pattern for both HMW-GS and LMW-GS (Figure 1A,B). In particular, HMW-GS (locus *Glu-B1*) is present with a band of 86 kDa (Bx7) and a band of 74 kDa with two isoforms (By8), while LMW-GS (locus *Glu-B3*) is represented by one main band of 42 kDa and minor bands (37, 32, 31 kDa) (LMW-2 profile) in accordance with previous studies [47,48]. This allelic combination was associated with favorable dough properties [37,49,50]. For gliadins, the pattern was composed by ω-gliadins lying in the 66−55 kDa range and the α-, β-, and γ-gliadins in the 44−33 kDa range (Figure 1C). The protein profiles were conserved in both cvs, but some differences were observed in their relative abundance in relation to both cv and year of cultivation, as shown by the densitometric analysis (Figure 2; Table S2). 

Regarding the HMW-GS (Figure 2A), the sub-units Bx7 and By8 showed significant variations (*p* < 0.05) in relation to the year (Y) and to the interaction between genotype and year (G × Y) (Table 3): in all environments, Bx7 was more abundant (*p* < 0.05) in 2017 than 2016 in both cvs, while By8 showed the opposite trend (Figure 2A). 

Although HMW-GS only accounts for up to 10% of total flour protein, it affects the gluten viscoelastic properties. The variation in the amounts of Bx and By sub-units in the different years could also have had an impact on grain quality [51]. In particular, the HMW-GS x-type seem to be more important than the y-type in determining dough properties [52]. The presence of the Bx7 subunit (which occurs in the greatest amount) is supposedly important for dough quality and loaf volume [53]. In our work, the higher abundance of Bx7 (Table S2) combined with that of LMW-GS in both cvs in 2017 suggests an improved quality of the flours. 

Regarding the LMW-GS (Figure 2B), there were significant variations for the 42 kDa subunit (*p* < 0.05) due to the combined effect of genotype and year, and for the 37 and 32 kDa subunits due to the year (Table 3). In Iride, LMW-42 and LMW-37 were more abundant in 2017 than in 2016, with the only exception of Quisquina. In Svevo, LMW-42 was more abundant in 2016 than in 2017, but not in Tolentino (Table S2). LMW-37 was more abundant in 2017 in all locations (Table S2). 

For Iride, LMW-32 was less abundant in all locations in 2017 than in 2016 except in Quisquina, while in Svevo, it was more abundant in Argelato and Foggia in the same year (Table S2). In agreement with our results, Giuliani and colleagues [37] reported that this protein is down-regulated under high temperatures in cv Svevo. 

The gliadin fraction was also quantified (Figure 2C); proteins of 44 and 41 kDa, corresponding to γ gliadins, were more abundant in 2016 than 2017 (*p* < 0.05) in both cvs and at all cultivation sites. Proteins of 34 and 33 kDa, corresponding to α and α/β gliadins, were more abundant in 2017 than 2016 (*p* < 0.05). In particular, the 33 kDa sub-unit was undetectable in SDS-PAGE gel in 2016 in Svevo (Table S2). The differences observed were related to the year of cultivation (Table 3). Similarly, a higher gliadin amount and gli/glu ratio were reported under environmental stress during grain filling, in particular, an increase in the α-gliadin fraction in Svevo under conditions of water stress during the early to mid stage of grain filling [37]. Several authors reported a down-regulation of γ-gliadins by high temperatures, identifying this protein as being sensitive to environmental changes [54,55]. 

#### 3.2.3. Analysis of Starch Granules 

**Figure 3:**
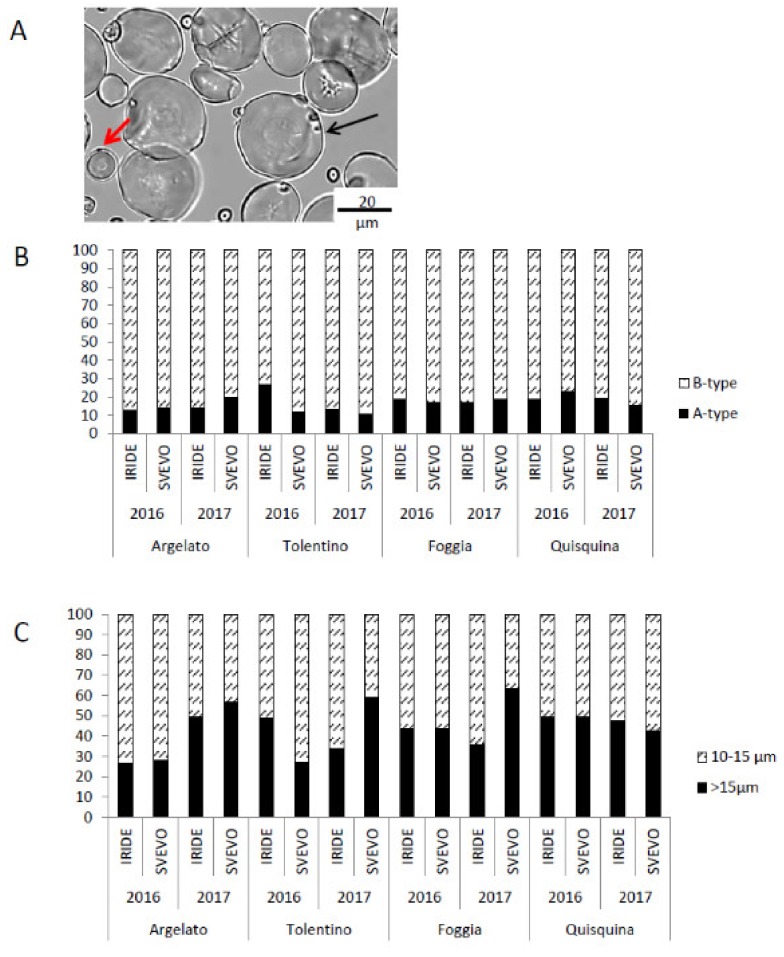
Starch granule analysis. (**A**) Light microscopy image of starch granules, example of granules obtained from Svevo cultivated in Tolentino in 2017. The black and red arrows indicate the A and B granules, respectively. The image is shown at the same level of magnification with 20 µm scale bars. (**B**) Quantification of A- and B-type starch granules (% of observed objects). (**C**) Size discrimination of A-type granules (% of total A-granules).

Optical microscopy was shown to be a useful way to study starch granules in terms of structure and distribution. In particular, we considered both A (diameter >10 µm) and B (diameter <10 µm) granules (Figure 3A): A-granules were less abundant than B-granules, even though they represented the majority of the starch biomass. The A-granules formed soon after anthesis and grew throughout grain filling, while the B-granules appeared some days after anthesis and remained considerably smaller. A-granules varied from 10.8% to 23%, while B-granules varied from 73.4% to 98.2%, although the differences were not significant in all samples (Figure 3B). In many samples, A-granules had diameters larger than 15 µm in 2017 (Figure 3C); in Svevo, these larger granules were two-fold more abundant in all locations as compared with 2016; in Iride, a similar result was observed only in the Argelato location (Figure 3C).

These results are in agreement with several studies showing that the environmental conditions may have a strong effect on the median size of A and B granules and also on the total amount of starch [11,56,57,58]. Moreover, high temperatures shortened the duration of starch accumulation [39].

Temperature stress is critical for starch accumulation [59]; high temperatures alter starch granule size, shape, and structure, and may also cause pitting and fissures, the extent of which depends on the severity and duration of stress [58]. Drought stress, associated usually with high temperatures, reduces the grain starch-lipid content and increases the pasting temperature, thus causing higher viscosity [18,19]. 

#### 3.2.4. Analysis of Polyphenols and Antioxidant Capacity 

The phenolic compositions of the investigated cvs are shown in Table 4. The three classes of free, soluble, conjugated, and bound phenolic acids represent different proportions of the total phenolic acids with significant variability in the amount of each class in relation to genotype, year, and site of cultivation (Table 3 and Table 4). Free phenolic acids and conjugated phenolic acids followed the same trend in both cvs. The amounts of these polyphenols were statistically different in all environments; free and conjugated phenolic acids showed the highest amounts in Quisquina: 11.89 and 104.95 µg GAE/g, respectively (Table 4). The variations depended on the year of cultivation but not on the genotype (Table 3). These results are in agreement with other studies which showed that the soluble form of polyphenols was particularly influenced by a combination of environmental factors [22,60]. 

Bound phenolic acids, which are the most abundant class, showed variation in relation to year, location, and genotype (Table 3); in particular, Svevo had a higher content than Iride (*p* < 0.05), and samples from the year 2017 had a higher content than samples from the year 2016 (*p* < 0.05). The highest amounts were observed in Foggia and Quisquina: 339.30 and 483.11 µg GAE/g, respectively. It has been previously reported that in wheat bran, the amount of polyphenols is highly correlated with the antioxidant activity [61]. Similarly, we observed a positive correlation between the polyphenol amount and ABTS^+^ scavenging capacity. Effectively, the highest ABTS^+^ scavenging capacity was observed in both cvs grown in Quisquina in all samples grown in 2017 (Table 3 and Table 4). This result may be explained by the higher temperatures and lower rainfall during grain filling measured in 2017 as compared to 2016 (Table S1). Therefore, higher stressing temperatures in 2017 may have increased radicals and oxidative species. Previous studies also provided evidence of the influences of genotype, growing area, and year of cultivation on the amount of phenolic acids and the antioxidant activity in wheat [14,23,62]. 

In this study, we characterized two wheat cvs, which are among the most cultivated in Italy for their medium to high yield combined with the good quality of the flour (Table 1). They performed differently in terms of gluten content and composition, starch accumulation, and antioxidant capacities, which are parameters that influence the quality and nutritional value of the flour. In particular, increases in the glu/gli ratio, LMW-GS content, polyphenols, antioxidant activity, and size of the starch A-granules were observed in 2017 with respect to 2016, probably due to the higher temperatures during grain filling. Our data support the high quality of these two Italian cvs, as evaluated by Italian crop quality reports in 2017.

## Figures and Tables

**Table 1:** Main agronomic traits and grain quality of durum wheat cultivars Iride and Svevo.

Name	Variety Characteristics	Plant Characteristics		Grain Quality		Tolerance	
Iride	Pedigree Altar 84 × Ionio	Seasonal Type	spring	Test Weight	high	Powdery Mildew	susceptible
	Release in 1996	Heading Time	early	Yellow index	medium	Leaf Rust	susceptible
	by PSB s.p.a.	Height	medium	Protein Content	medium (>12%)	Septoria	susceptible
		Awn color	black	Gluten quality	83%	Abiotic stress	excellent
		Potential yield	high	Glu A1	null	Lodging	good
				Glu B1	7+8		
				Glu B3	LMW-2		
Svevo	Pedigree Cimmyt line × Zenit	Seasonal Type	spring	Test Weight	good	Powdery Mildew	susceptible
	Release in 1996	Heading Time	early	Yellow index	high	Leaf Rust	susceptible
	by PSB s.p.a.	Height	medium-high	Protein Content	good (>14%)	Septoria	tolerant
		Awn color	whitish	Gluten quality	85%	Abiotic stress	excellent
		Potential yield	medium	GluA1	null	Lodging	good
				Glu B1	7+8		
				GluB3	LMW-2		

**Table 2:** Mean values of grain protein content and gluten fractions of Iride and Svevo cultivated in 2016 and 2017 in four Italian locations.

		GPC%	Gliadins %	HMW-GS %	LMW-GS %	glu/gli	HMW-GS/LMW-GS
Location *	Argelato	13.63 ± 1.35^b^	47.27 ± 5.64^a^	24.37 ± 3.22^a^	28.36 ± 6.50^a^	1.14 ± 0.25^a^	1.02 ± 0.40^a^
	Tolentino	13.80 ± 1.10^b^	46.72 ± 5.63^a^	25.04 ± 0.80^a^	28.24 ± 5.46^a^	1.16 ± 0.26^a^	0.91 ± 0.18^a^
	Foggia	13.28 ± 0.85^b^	47.23 ± 6.04^a^	25.23 ± 2.41^a^	26.75 ± 7.75^a^	1.14 ± 0.28^a^	0.99 ± 0.37^a^
	Quisquina	15.33 ±1.00^a^	44.87 ± 6.98^a^	26.32 ± 1.71^a^	28.81 ± 6.41^a^	1.27 ± 0.35^a^	0.95 ± 0.22^a^
Year ^†^	2016	13.99 ± 1.56^a^	51.67 ± 1.09^a^	26.21 ± 1.62^a^	22.12 ± 2.40^b^	0.93 ± 0.04^b^	1.20 ± 0.19^a^
	2017	14.03 ± 1.00^a^	41.38 ± 2.09^b^	24.26 ± 2.78^a^	34.36 ± 1.99^a^	1.42 ± 0.12^a^	0.73 ± 0.07^b^
Cultivar ^‡^	Iride	13.35 ± 1.01^b^	46.53 ± 6.14^a^	25.89 ± 2.97^a^	27.58 ± 7.71^a^	1.18 ± 0.29^a^	1.05 ± 0.31^a^
	Svevo	14.66 ± 1.19^a^	46.52 ± 5.31^a^	24.59 ± 1.66^a^	28.90 ± 5.90^a^	1.17 ± 0.26^a^	0.89 ± 0.23^a^

Different letters in the same column, for each cultivar in each year, correspond to statistically different values (*p* < 0.05 one way ANOVA, post hoc Dunn’s test). * for each location, mean values of the two cvs in the two years; ^†^ for each year, mean values of the two cvs in the four locations; ^‡^ for each cultivar, mean values of the four locations in the two years.

**Table 3:** Results of a multivariate analysis evaluating differences for the amount of grain protein content, gluten protein sub-units, phenolic compounds, and antioxidant properties.

Trait	Source of Variation		Df ^a^	SS ^b^	MS ^c^	F	*p*-Value
Gluten Fraction	Gliadins	Year (Y)	1	423.742	423.742	147.774	0.000
	LMW	(Pillai’s Trace = 0.000)	1	522.923	522.923	194.221	0.000
Gluten protein Bands %	LMW 42	Genotype (G) (Pillai’s Trace = 0.050)	1	123.099	123.09	7.467	0.018
	GLY 44		1	57.646	57.646	5.065	0.044
	GLY 41		1	36.754	36.754	6.829	0.023
	GLY 32-34		1	94.868	94.868	19.006	0.001
	B_x	Year (Pillai’s Trace = 0.000)	1	428.076	428.076	35.268	0.000
	B_y		1	428.076	428.076	35.268	0.000
	LMW 37		1	102.061	102.061	11.045	0.006
	LMW 32		1	41.602	41.602	4.865	0.048
	GLY 44		1	1497.497	1497.497	131.568	0.000
	GLY 41		1	332.971	332.971	61.863	0.000
	GLY 32-34		1	515.971	515.971	103.369	0.000
	GLY 28-30		1	718.240	718.240	158.343	0.000
	B_x	G × Y (Pillai’s Trace = 0.036)	1	58.523	58.523	4.822	0.049
	B_y		1	58.522	58.522	4.822	0.049
	LMW 42		1	81.000	81.000	4.914	0.047
	GLY 41		1	45.327	45.327	8.421	0.013
Polyphenols antioxidant	Free	Environment (E) (Pillai’s Trace = 0.000)	3	143.130	47.710	291.553	0.000
	Conjugates		3	14075.706	4691.902	159.130	0.000
	Bounds		3	299366.536	99788.845	1150.315	0.000
	TEAC		3	1953.443	651.148	1708.501	0.000
	Free	G × Y (Pillai’s Trace = 0.000)	3	46.173	15.391	94.053	0.000
	Conjugates		3	4113.264	1371.088	46.502	0.000
	Bounds		3	280728.063	93576.021	1078.697	0.000
	TEAC		3	23288.355	7762.785	20368.233	0.000
	Free	G × Y × E (Pillai’s Trace = 0.000)	9	52.134	5.793	35.399	0.000
	Conjugates		9	5665.122	629.458	21.349	0.000
	Bounds		9	296814.790	32979.421	380.170	0.000
	TEAC		9	4783.027	531.447	1394.428	0.000

^a^ Degree of freedom; ^b^ Sum of Squares; ^c^ mean square.

**Table 4:** Mean values of the antioxidant properties for wheat cultivars, years, and locations.

		Free Phenolics (µg GAE/g)	Conjugated Phenolics (µg GAE/g)	Bounds Phenolics (µg GAE/g)	Antioxidant Capacity TEAC (µM TE/g)
Location *	Argelato	8.19 ± 0.12^c^	67.94 ± 0.23^c^	288.48 ± 13.95^c^	61.40 ± 0.99^b^
	Tolentino	7.46 ± 0.11^d^	60.72 ± 0.22^d^	292.26 ± 7.60^c^	45.47 ± 0.68^d^
	Foggia	10.23 ± 0.59^b^	85.75 ± 0.29^b^	339.30 ± 13.86^b^	58.41 ± 0.76^c^
	Quisquina	11.89 ± 0.35^a^	104.95 ± 0.36^a^	483.11 ± 6.92^a^	62.75 ± 0.42^a^
Year ^†^	2016	8.65 ± 0.18^b^	72.54 ± 0.25^b^	303.22 ± 10.42^b^	38.83 ± 0.98^b^
	2017	10.18 ± 0.41^a^	87.14 ± 0.30^a^	398.36 ± 10.75^a^	75.19 ± 0.45^a^
Cultivar ^‡^	Iride	9.47 ± 0.40^a^	80.06 ± 0.28^a^	294.03 ± 10.91^b^	65.25 ± 0.78^a^
	Svevo	9.35 ± 0.18^a^	79.62 ± 0.27^a^	407.55 ± 10.26^a^	48.73 ± 0.65^b^

Different letters in the same column, within location, year and cultivar correspond to statistically different values (*p* < 0.05 one way ANOVA, post hoc Dunn’s test). * for each location, mean values of the two cvs in the two years; ^†^ for each year, mean values of the two cvs in the four locations; ^‡^ for each cultivar, mean values of the four locations in the two years.

## Supplementary Materials

The following are available online at https://www.mdpi.com/2304-8158/9/3/315/s1, Table S1: Meteorological conditions observed in the four locations in 2016 and 2017. Table S2: Densitometry analysis of gluten fractions.
