# Supplementary material for: Proteins and Metabolites as Indicators of Flours Quality and Nutritional Properties of Two Durum Wheat Varieties Grown in Different Italian Locations
Source: Foods. 2020 Mar 9;9(3):315. doi: 10.3390/foods9030315 (PMC7143883; doi:10.3390/foods9030315)
Supplement: Supplementary file 1 [file foods-09-00315-s001.pdf]

Supplementary material

Table S1. Meteorological conditions observed in the four locations in 2016 and 2017. The average amount of rainfall and the minimum and maximum temperatures are reported on a quarterly basis from sowing to harvest.

Year	Site	Location (Lat Long, Alt)	Rainfall (Average mm) Nov–Jan	Feb–Apr	May–Jun	Temperature (T_{min}–T_{max} °C) Nov–Jan min	Nov–Jan max	Feb–Apr min	Feb–Apr max	May–Jun min	May–Jun max
2016	Argelato (BO)	44°39′ N, 11°20′ E, 25 m	16.5	41.2	52.3	3.2	11.2	7.1	16.6	16.1	24.9
	Tolentino (MC)	43°14′ N, 13°24′ E, 184 m	8.9	27.9	37.1	5.9	12.2	9.3	17.2	15.8	25.0
	Foggia (FG)	41°27′ N, 15°30′ E, 76 m	10.4	19.4	29.0	7.4	10.3	8.6	17.6	14.4	20.2
	S. Stefano Quisquina (AG)	37°32′ N, 13°31′ E, 180 m	10.8	13.0	4.8	8.2	16.4	9.5	15.5	14.6	24.3
2017	Argelato (BO)	44°39′ N, 11°20′ E, 25 m	14.3	15.7	23.3	−5	12.5	6.0	20.0	18.0	30.0
	Tolentino (MC)	43°14′ N, 13°24′ E, 184 m	16.0	26.9	15.3	−1	12.2	6.8	18.0	15.4	27.0
	Foggia (FG)	41°27′ N, 15°30′ E, 76 m	17.0	13.0	13.3	−1	12.4	9.3	18.0	17.0	32.0
	S. Stefano Quisquina (AG)	37°32′ N, 13°31′ E, 180 m	60.0	30.0	2.3	8.2	17.0	8.0	22.1	14.0	36.0

Table S2. Densitometry analysis of gluten fractions of Iride and Svevo cultivated in 2016 and 2017 in four Italian regions.

Cultivar	Fraction	Band range %	Argelato (Emilia-Romagna)		Tolentino (Marche)		Foggia (Puglia)		Quisquina (Sicily)	
			2016	2017	2016	2017	2016	2017	2016	2017
Iride	HMW	Bx_82.5 kDa	74.00	80.39	75.67	80.95	71.81	79.83	74.15	80.54
		By_74.5 kDa	26.00	19.61	24.33	19.05	28.19	20.17	25.85	19.46
	LMW	42 kDa	46.65	58.59	55.34	58.14	54.55	59.16	52.11	52.19
		37 kDa	28.04	30.15	27.74	28.07	21.01	31.25	24.79	30.05
		32 kDa	17.44	7.30	8.92	5.09	15.35	6.53	8.26	10.99
		31 kDa	7.87	3.97	8.00	8.70	9.08	3.06	14.84	6.75
	Gliadins	44 kDa	22.92	3.64	17.46	1.31	23.63	3.50	22.53	9.70
		41 kDa	15.20	8.57	14.93	10.24	17.04	10.97	19.05	13.41
		34 kDa	39.46	28.35	45.23	26.46	37.78	26.75	36.57	24.91
		33 kDa	2.30	16.14	3.06	12.06	3.93	17.98	2.91	13.09
Svevo	HMW	Bx_82.5 kDa	68.05	82.35	65.05	90.14	75.70	78.53	67.24	81.70
		By_74.5 kDa	31.95	17.65	34.95	9.86	24.30	21.47	32.76	18.30
	LMW	42 kDa	60.81	50.26	58.78	63.44	64.48	58.47	64.77	60.02
		37 kDa	28.29	35.21	21.09	30.14	24.08	28.74	22.51	29.61
		32 kDa	6.72	8.10	10.69	3.46	5.19	8.67	6.47	5.83
		31 kDa	4.19	6.44	9.44	2.96	6.26	4.12	6.25	4.54
	Gliadins	44 kDa	28.93	2.57	26.76	8.17	27.87	11.96	27.17	1.63
		41 kDa	19.44	13.04	21.68	8.05	24.21	9.65	26.48	11.11
		34 kDa	33.05	24.18	33.43	23.24	35.09	23.61	30.85	23.10
		33 kDa	0.00	15.75	0.00	10.39	0.00	16.30	0.00	17.69
